# An *Alu*-Based Phylogeny of Lemurs (Infraorder: Lemuriformes)

**DOI:** 10.1371/journal.pone.0044035

**Published:** 2012-08-28

**Authors:** Adam T. McLain, Thomas J. Meyer, Christopher Faulk, Scott W. Herke, J. Michael Oldenburg, Matthew G. Bourgeois, Camille F. Abshire, Christian Roos, Mark A. Batzer

**Affiliations:** 1 Department of Biological Sciences, Louisiana State University, Baton Rouge, Louisiana, United States of America; 2 Department of Behavioral Neuroscience, Oregon Health & Science University, Portland, Oregon, United States of America; 3 Department of Environmental Health Sciences, University of Michigan, Ann Arbor, Michigan, United States of America; 4 Department of Molecular and Cellular Physiology, Louisiana State University Health Sciences Center, Shreveport, Louisiana, United States of America; 5 Gene Bank of Primates and Primate Genetics Laboratory, German Primate Center, Göttingen, Germany; Texas A&M University, United States of America

## Abstract

Lemurs (infraorder: Lemuriformes) are a radiation of strepsirrhine primates endemic to the island of Madagascar. As of 2012, 101 lemur species, divided among five families, have been described. Genetic and morphological evidence indicates all species are descended from a common ancestor that arrived in Madagascar ∼55–60 million years ago (mya). Phylogenetic relationships in this species-rich infraorder have been the subject of debate. Here we use *Alu* elements, a family of primate-specific Short INterspersed Elements (SINEs), to construct a phylogeny of infraorder Lemuriformes. *Alu* elements are particularly useful SINEs for the purpose of phylogeny reconstruction because they are identical by descent and confounding events between loci are easily resolved by sequencing. The genome of the grey mouse lemur (*Microcebus murinus*) was computationally assayed for synapomorphic *Alu* elements. Those that were identified as Lemuriformes-specific were analyzed against other available primate genomes for orthologous sequence in which to design primers for PCR (polymerase chain reaction) verification. A primate phylogenetic panel of 24 species, including 22 lemur species from all five families, was examined for the presence/absence of 138 *Alu* elements via PCR to establish relationships among species. Of these, 111 were phylogenetically informative. A phylogenetic tree was generated based on the results of this analysis. We demonstrate strong support for the monophyly of Lemuriformes to the exclusion of other primates, with Daubentoniidae, the aye-aye, as the basal lineage within the infraorder. Our results also suggest Lepilemuridae as a sister lineage to Cheirogaleidae, and Indriidae as sister to Lemuridae. Among the Cheirogaleidae, we show strong support for *Microcebus* and *Mirza* as sister genera, with *Cheirogaleus* the sister lineage to both. Our results also support the monophyly of the Lemuridae. Within Lemuridae we place *Lemur* and *Hapalemur* together to the exclusion of *Eulemur* and *Varecia*, with *Varecia* the sister lineage to the other three genera.

## Introduction

Lemurs (infraorder: Lemuriformes) are an ecologically and phenotypically diverse radiation of strepsirrhine primates endemic to the island of Madagascar. Varying in size from the tiny mouse lemurs (*Microcebus*), the smallest living primates at 23–29 cm, to the indris (*Indri*) at 64–72 cm, lemurs display stunning diversity in length, weight, diet, behavior, and pelage [1]. The prevailing genetic and morphological evidence supports the monophyletic descent of all extant and extinct lemur species from a common ancestor that arrived on Madagascar between 55–60 million years ago. The ancestor most likely dispersed via a rafting event across the Mozambique Channel from the African mainland [2], [3], [4], [5], [6]. In the ensuing period lemurs have diversified to occupy a wide array of ecological niches across Madagascar. At present five families and approximately 100 species are recognized (the number of species differs depending upon the author(s) consulted), with dozens of new species discovered or elevated from subspecies status since the 1980s. In the first decade of the 21st century alone researchers described 41 new lemur species [1], [7]. Concerns have been raised regarding overzealousness in the description of new species, but it cannot be disputed that lemurs are a much more speciose radiation than they were thought to be a few decades ago [8].

A broad review of extant species in 2010 [1] recognized 101 species of lemur in 15 genera grouped into five families: Cheirogaleidae, the mouse, dwarf, and fork-marked lemurs, with 31 species in five genera (*Allocebus*, *Cheirogaleus*, *Microcebus*, *Mirza*, and *Phaner*); Daubentoniidae, the aye-aye, with one species in one genus (*Daubentonia*); Indriidae, the indris, sifakas, and wooly lemurs, with 19 species in three genera (*Avahi*, *Indri*, and *Propithecus*); Lemuridae, the ring-tailed, brown (or true), ruffled, and bamboo (or gentle) lemurs, with 24 species in five genera (*Eulemur*, *Hapalemur*, *Lemur*, *Prolemur*, and *Varecia*); and Lepilemuridae, the sportive lemurs, with 26 species in one genus (*Lepilemur*). There are, in addition, 17 known species of extinct lemurs classified into eight genera and three families. Many of these extinctions are recent and related to the arrival of humans on Madagascar ∼2kya [1], [9].

Lemur phylogeny remains controversial at all taxonomic levels, and a number of studies have been conducted over the last four decades using various approaches, including morphology [10], [11], [12], karyotyping [13], [14], mitochondrial DNA analysis [15], nuclear DNA [3], [16], [17], [18], combinations of mitochondrial and nuclear markers [4], combinations of molecular and morphological characters [19], retrotransposon analysis [3], [20], and the genetic and morphological relationships of parasites across species [21]. The large body of literature on lemur phylogeny is in agreement on several key points, including the monophyly of the infraorder and the grouping of lemurs alongside the infraorder Lorisiformes within the strepsirrhine clade, the tooth-combed primates, as sister taxa to all other living primates [17], [18]. Additionally, it is generally agreed that the family Daubentoniidae is the basal lineage within the lemuriforms, having been first to diverge from a shared common ancestor with the other four extant families [22]. Because of its extreme temporal divergence and phenotypic variation from other lemurs the family Daubentoniidae is sometimes separated in its own infraorder, Chiromyiformes [3], [18], [23].

Outside of these points of agreement are the contentious relationships among the four remaining families. Two competing family-level phylogenies have emerged during the past decade. Both agree on the basal position of Daubentoniidae to the other four families. Horvath et al. (2008) [17] place the Indriidae, Cheirogaleidae, and Lepilemuridae together, and all three as basal to Lemuridae, a position supported by Yoder (1997) [24], Pastorini, Thalmann, and Martin (2003) [16], and Yoder and Yang (2004) [4]. Alternatively, et al. (2008) [25], in an analysis that also included subfossil lemurs, placed Indriidae and Lemuridae together to the exclusion of Cheirogaleidae and Lepilemuridae, a position supported by Delpero at al. (2001) [15], Roos, Schmitz, and Zischler (2004) [3], and Bochkob et al. (2011) [21]. These conflicting phylogenies rest primarily on the position of Indriidae, and whether this family represents a basal lineage to Lemuridae or is of more recent divergence, with Cheirogaleidae and Lepilemuridae basal to both. We use *Alu* elements, a family of primate-specific mobile elements, to resolve these conflicting phylogenetic analyses.

SINEs (Short INterspersed Elements) are a class of non-autonomous retrotransposons of <500 base pairs (bp) length that use RNA intermediaries to copy and insert themselves elsewhere within host genomes [26], [27], [28], [29]. SINEs are particularly useful genetic markers in the establishment of evolutionary relationships for several reasons. First, they are nearly-homoplasy-free markers [30], [31]. The ancestral state is known to be the absence of the element, and each new element to arise is a distinct evolutionary event within a lineage. Thus, individuals sharing the same SINE at an orthologous locus are thought to be of common ancestry [29], [30], [31], [32], [33], [34], [35], [36]. Second, once a SINE has inserted into a genome it is very rarely precisely excised. Thirdly, SINEs are relatively easy to evaluate using a locus specific PCR assay, making them potentially useful markers for conservationists [30].

The use of SINEs as evolutionary and phylogenetic markers was first applied nearly two decades ago to resolve phylogenetic relationships among fish species [32]. Since this early work the reliability of SINEs as phylogenetic markers has been well documented across many species, and the *Alu* family of primate-specific SINEs has been demonstrated to be particularly useful at elucidating phylogenetic relationships between primate species [3], [20], [36], [37], [38], [39], [40], [41], [42], [43], [44], [45], [46].

**Figure 1:**
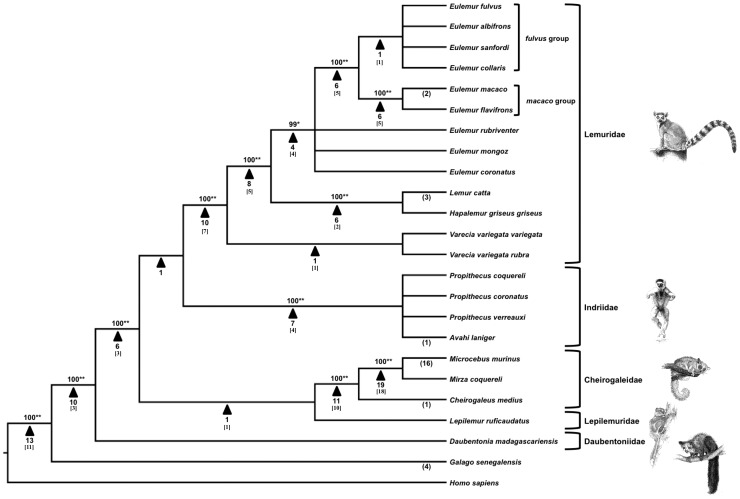
The most parsimonious tree generated from analysis of 138 *Alu* insertions in Lemuriformes. The amplification patterns of the *Alu* insertions were used to construct a Dollo parsimony tree of phylogenetic relationships with *G. senegalensis* and *H. sapiens* as outgroups using the MESQUITE and PAUP* programs. Numbers above branches are bootstrap values. The significance level of each node supported by insertions as determined by likelihood testing is indicated by either *(p<0.05) or **(p<0.01). Numbers below arrows indicate the number of unambiguous loci supporting that node. Numbers in brackets below arrows indicate the number of loci at a given informative node identified by McLain et al. (2012). Numbers in parentheses represent insertions that are only present in one species or group. These insertions are not parsimony-informative. Consistency index (CI): 1.000; Homoplasy index (HI): 0.000; Retention index (RI): 1.000.

*Alu* elements are a SINE of ∼300 bp length found only in primate genomes. Originally derived from 7SL RNA in a common ancestor of all living primates, *Alu* elements have propagated to the point where they comprise a significant component of primate genomes [47], [48], [49]. *Alu* elements are classified into subfamilies, with *Alu*J being the oldest and therefore present in the genomes of all living primates [29], [50]. Younger lineage-specific subfamilies exist across the primate radiation, with some subfamilies presently active and others no longer producing new copies or subfamilies [29]. Liu et al. (2009) [51] assigned the subfamily designation *Alu*L to elements found in Lemuriformes. Earlier studies have examined aspects of lemur phylogeny using SINEs. Roos, Schmitz, and Zischler (2004) [3] used a combination of SINE and mitochondrial markers to reconstruct a phylogeny of the strepsirrhine radiation, while Herke et al. (2007) [20] examined relationships among some lemur species as part of a larger study involving the building of an *Alu*-based key for primate species identification. However, no exclusively *Alu*-based phylogeny focused on this infraorder has ever been reported. Here, using a combination of computational methods, PCR display methodology, and DNA sequencing, we use 138 *Alu* insertions specific to the Malagasy strepsirrhine lineage, including 22 loci previously reported by Herke et al. (2007) [20] and 17 loci previously reported by Roos, Schmitz, and Zischler (2004) [3], to construct a phylogeny of Lemuriformes (Fig. 1).

## Materials and Methods

### 1. Computational Methodology

Genomic sequence of the grey mouse lemur (*Microcebus murinus*; GenBank accession number ABDC01000000) generated at 1.9X coverage by the Broad Institute for the 29 Mammals Project was obtained in the form of 669,735 genomic contigs at 1.6 Gb total length [52]. The majority of our PCR primers were designed using sequence from the *Microcebus murinus* genome. Additionally, genomic sequence was obtained from GenBank for the following species: *Lemur catta* (ring-tailed lemur), *Eulemur macaco* (black lemur), *Eulemur coronatus* (crowned lemur), *Propithecus coquereli* (Coquerel’s sifaka), *Daubentonia madagascariensis* (aye-aye), and *Cheirogaleus medius* (fat-tailed dwarf lemur). Sequences for these species were searched for putative lemur-specific *Alu* insertions based upon seven previously identified *Alu*L consensus sequences [51] using an in-house installation of the RepeatMasker program [53] with a custom library.

In-house Perl scripts were used to parse the RepeatMasker output for easier examination. Elements identified by RepeatMasker in *Microcebus murinus* genomic contigs as members of an *Alu*L subfamily and >280 bp in length were compared to four non-lemuriform primate genomes, human (hg19), chimpanzee (panTro2), orangutan (ponAbe2), and rhesus macaque (rheMac2), using the BLAST-Like Alignment Tool (BLAT) available at http://genome.ucsc.edu [53]. Elements found to be absent in these outgroups, and that had orthologous flanking sequence that would allow primer design were marked for further examination. In the case of putative Lemuriformes-specific *Alu* elements obtained from other lemur species via GenBank, the Ensembl BLAT tool was used to compare the sequence to the genomic data of *Microcebus murinus* [54]. This was done to differentiate between *Alu* subfamilies distinct to particular lemuriform lineages and more effectively locate subfamilies specific to particular genera. The CLC Main Workbench v.5 software suite was used to align sequences and identify regions suitable for primer building (http://www.clcbio.com/index.php?id=92). Oligonucleotide primers for PCR assay were designed in the regions flanking the element using the Primer3Plus program [55]. These primers were tested computationally against available primate genomes using the *in-silico* PCR tool on the UCSC Genome Bioinformatics website. Additionally, 22 primer pairs from Herke et al. (2007) [20] and 17 primer pairs from Roos, Schmitz, and Zischler (2004) [3] were added to our analysis to provide additional resolution of phylogenetic relationships among the Lemuriformes (See Table S1).

### 2. PCR and DNA Sequencing

Primers were tested for amplification with lemur DNA templates corresponding to the species in which the primers were designed using a temperature gradient PCR (48°–65°C) to determine the proper annealing temperature for analysis of non-human samples. All loci were screened on a primate panel composed of *Homo sapiens* (HeLa) genomic DNA and samples from 23 strepsirrhine primates, including the 22 lemur species listed in Table 1, and one out-group (non-Lemuriformes) strepsirrhine primate, *Galago senegalensis* (Senegal bushbaby). For species with limited amounts of genomic DNA available, the samples were subjected to whole genome pre-amplification using the GenomiPhi genome amplification kit (Amersham, Sunnyvale, CA).

**Table 1:** DNA samples of all species examined in this study.

Species Names	Common Names	Origin	ID Number
*Lemur catta*	Ring-tailed lemur	Coriell^a^	NG07099
*Eulemur coronatus*	Crowned lemur, “Bes”	DLC^b^	6251m
*Eulemur albifrons*	Brown (white-fronted) lemur	IPBIR^c^	PR00245
*Eulemur collaris*	Collared brown lemur, “Andre”	DLC^b^	4545m
*Eulemur fulvus*	Brown lemur, “Globin”	DLC^b^	3562f
*Eulemur sanfordi*	Sanford’s brown lemur, “Beby”	DLC^b^	6098f
*Eulemur macaco*	Macaco black lemur	IPBIR^c^	PR00266
*Eulemur flavifrons*	Blue-eyed black lemur, “Lange”	DLC^b^	6521f
*Eulemur mongoz*	Mongoose lemur, “Esperanza”	DLC^b^	5717f
*Eulemur rubriventer*	Red-bellied lemur, “Paiute”	DLC^b^	6559m
*Hapalemur griseus griseus*	Lesser bamboo lemur, “Beamish”	DLC^b^	1359m
*Varecia variegata rubra*	Red ruffed lemur, “Dembowska”	DLC^b^	6424f
*Varecia variegata variegata*	Black and white ruffed lemur, “Bopp”	DLC^b^	6720m
*Microcebus murinus*	Gray mouse lemur	SDFZ^d^	KB6993
*Mirza coquereli*	Coquerel’s mouse lemur	IPBIR^c^	PR00871
*Cheirogaleus medius*	Fat-tailed dwarf lemur	IPBIR^c^	PR00794
*Lepilemur ruficaudatus*	Red-tailed sportive lemur	GBP^e^	N/A
*Avahi laniger*	Wooly lemur	GBP^e^	AL
*Propithecus coquereli*	Coquerel’s sifaka	DLC^b^	6723f
*Propithecus coronatus*	Van der Decken’s Sifaka	GBP^e^	PC495
*Propithecus verreauxi*	White sifaka	GBP^e^	PV760
*Daubentonia madagascariensis*	Aye-aye, “Annabel Lee”	DLC^b^	6262f
*Galago senegalensis*	Senegal bushbaby	Batzer^f^	PR01035
*Homo sapiens*	Human, HeLa	ATCC^g^	CCL-2

a Coriell Institute for Medical Research, 403 Haddon Avenue, Camden, NJ 08103, USA.

b Duke Lemur Center (DLC), Duke University, Durham, NC 27708, USA.

c Integrated Primate Biomaterials and Information Resource (IPBIR), http://ccr.coriell.org/Sections/Collections/.

d Frozen Zoo, San Diego Zoo (SDFZ), http://conservationandscience.org.

e Gene Bank of Primates (GBP), German Primate Center, Göttingen, Germany.

f Batzer: Adenovirus 12 SV40-transformed fibroblasts maintained in the lab of Dr. Mark Batzer.

g From cell lines provided by American Type Culture Collection (ATCC), P.O. Box 1549, Manassas, VA 20108, USA.

DNA samples of all species examined in this study.

**Figure 2:**
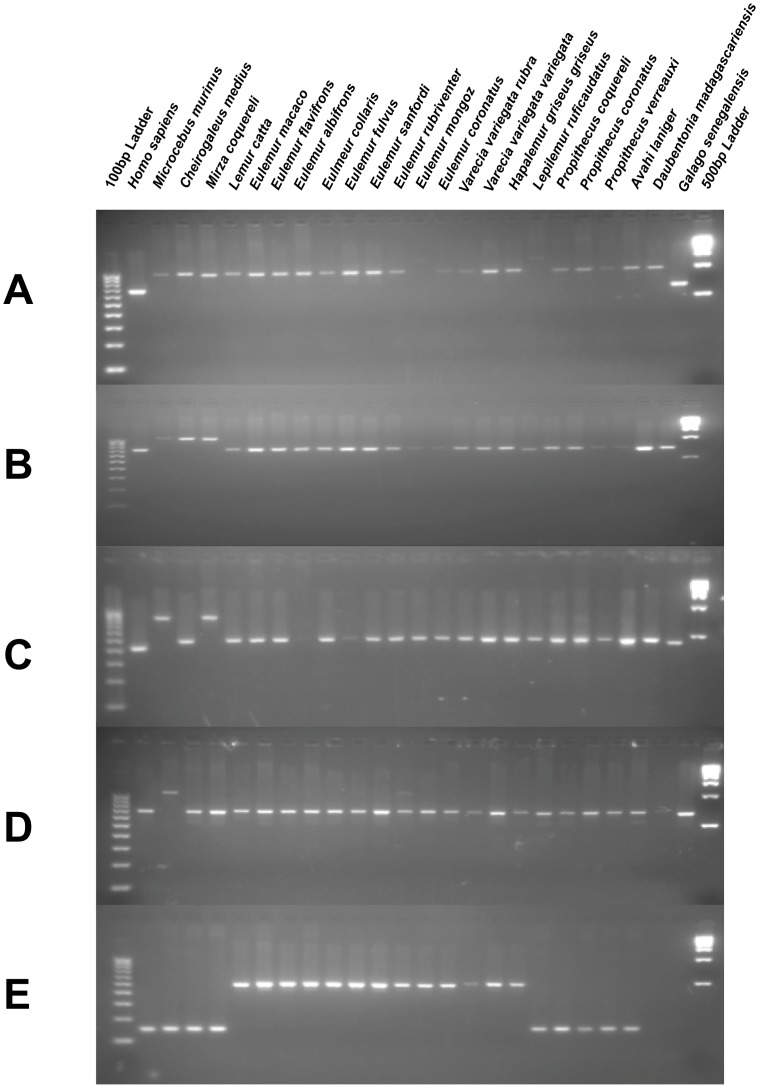
PCR amplification of polymorphic *Alu* insertions in Lemuriformes. Gel photographs displaying the methodology for establishing evolutionary relationships using *Alu* elements. The presence and absence of elements, supplemented by sequencing to eliminate the possibility of confounding events, is used to determine which species are more closely related. A total of 5 gel electrophoresis results on a 24-species primate panel are shown with *H. sapiens* and *G. senegalensis* as outgroups. **A:** Amplification of locus Str71B, an *Alu* insertion shared by the infraorder Lemuriformes. **B:** Amplification of locus MmA39, an *Alu* insertion shared by the family Cheirogaleidae. **C:** Amplification of locus MmA27, an *Alu* insertion shared by the sister genera *Microcebus* and *Mirza*. **D:** Amplification of locus Str59, an *Alu* insertion specific to the genus *Microcebus*. **E:** Amplification of locus Em6, an *Alu* insertion affirming the monophyly of the family Lemuridae to the exclusion of other lemur species and outgroups.

PCR amplification of each locus was performed in 25 µl reactions using 15 ng of template DNA, 200 nM of each primer, 200 µM dNTPs in 50 mM KCl, 1.5 mM MgCl_2_, 10 mM Tris-HCl (pH 8.4), and 2 units of *Taq* DNA polymerase. PCR reaction conditions were as follows: an initial denaturation at 95°C for 1 minute, followed by 32 cycles of denaturation at 95°C, annealing at the previously determined optimal annealing temperature, and extension at 72°C for 30 seconds each, followed by a final extension of 72°C for 1 minute. PCR products were analyzed on 2% agarose gels stained with 0.25 ug ethidium bromide and visualized with UV fluorescence (Fig. 2). A list of all loci, corresponding primer pairs, and optimal annealing temperatures for each are available as Table S1 for this study.

In the case of loci exhibiting relationships inconsistent with the most parsimonious tree, DNA cloning and sequencing was performed to verify the subfamily identity of the element in each species, in order to be certain that the relationships ascertained visually from PCR and gel electrophoresis did in fact share sequence identity. Gel slices were cut and purified using the Wizard SV Gel and PCR Clean-Up System (www.promega.com). Products from the individual PCRs were then cloned using the TOPO-TA cloning kit (Invitrogen, Carlsbad, CA) and sequenced using chain termination sequencing on an ABI 3130 Genetic Analyzer [56]. The resulting sequence data was then aligned and examined to determine if the locus in question was, in fact, the same element across multiple species, or a confounding event such as a parallel independent insertion or independent lineage sorting [32], [34].

### 3. Phylogenetic Analysis

A Dollo parsimony matrix was created using MESQUITE 2.75, a multi-faceted analysis program for applications in evolutionary biology [57] (See Table S2). Dollo parsimony is particularly suited to *Alu*-based phylogenies because *Alu* elements are synapomorphic, presence-absence characters and nearly homoplasy-free genetic markers [41], [42], [43]. All loci were set to Dollo.up for parsimony analysis. If an *Alu* insertion was found to be present via PCR assay it was coded as “1” for the given locus in the matrix. If the insertion was absent it was coded as “0”. Loci that could not be resolved for a given species at a certain locus were coded with a “?”. The PAUP* version 4.0b10 software [58] was then used to perform a heuristic search on the data. A total of 10,000 bootstrap replicates were performed and a statistical test for evaluating SINE insertions based on a likelihood model [59] was used to assess the statistical significance of each branch on the resulting tree. The tree was then visualized using FigTree (http://tree.bio.ed.ac.uk/software/figtree/).

## Results and Discussion

### 1. Computational Data Mining in the Microcebus Murinus Genome

RepeatMasker identified a total of 294,218 putative *Alu* elements in the genome of *Microcebus murinus*, of which 229,774 were identified as belonging to the *Alu*L subfamily based on our consensus sequence. Another 16,224 *Alu*s were identified as members of the ancient *Alu*J subfamilies, with 3,169 identified as *Alu*Jb and 13,055 identified as *Alu*Jo, respectively. Churakov et al. (2010) [50] examined the *Alu*Jb subfamily in Strepsirrhines and found that it was likely inactive. Therefore the putative *Alu*Jbs identified in our RepeatMasker run were probably wrongly annotated by the program. Caution must be used when examining the total number of *Alu* elements identified, as the *Microcebus murinus* genome is unassembled and there are certainly contigs in our analysis that overlap. We guarded against examining the same locus twice by reviewing all loci in alignments to determine that they were, in fact, unique. Other elements were identified as belonging to various *Alu* subfamilies to which it is evolutionarily impossible for them to belong, such as *Alu*Y, a catarrhine-specific subfamily. These loci require additional examination for confirmation, but are likely members of Lemuriformes-specific subfamilies that are as-yet undocumented and were therefore not included in our custom RepeatMasker library. We focused on putative *Alu*L loci with sufficient orthologous flanking in available primate genomes in order to build the most optimal primers for elucidating phylogenetic relationships among lemur species. The large divergence time between the most recent common ancestor of the Lemuriformes and any other assembled primate genomes available for analysis necessitated the examination of a large number of potentially informative *Alu*L sites in order to construct functional primers. Sequence data generated from this project has been deposited in GenBank under the accession numbers (JX22863-JX228922; JX195193-JX195195; JX195187-JX195189). Sequence data for primers generated by Roos, Schmitz, and Zischler (2004) [3] is available from GenBank under the accession numbers (AY441478–AY441759). Sequence data for primers generated by Herke et al. (2007) [20] is available from GenBank under the accession numbers (DQ822046–DQ822070 and DQ843660–DQ843663).

### 2. Phylogeny of the Lemuriformes

We identified 138 *Alu* insertion loci in multiple species of the five lemuriform families. 111 of these were phylogenetically informative, and 27 were autapomorphic insertions. The majority of primer design was completed in the available nuclear DNA sequence from the *Microcebus murinus* genome. In the case of PCR amplifications suggesting a branching pattern different from that of the majority of loci, sequencing universally revealed either a near-parallel independent insertion or another type of insertion/deletion event not affecting the topology of the tree. Loci that did not challenge the majority of loci at a node or did not display an incongruous pattern of relationships within the Lemuriformes were not subjected to sequencing. It is possible, but unlikely, that this had an effect of the topology of the tree. Most of the loci included in this study were identified computationally in lemur nuclear DNA sequence available via GenBank. The analysis of these loci resulted in a single most parsimonious tree (Fig. 1; CI = 1.000; HI = 0.0000; RI = 1.000). With the exception of two branches of our tree, discussed in more detail below, every clade was robustly supported (p-value <0.05) under the maximum likelihood test developed in Waddell et al. (2001) [59]. The branches also had high levels of support based upon bootstrap analysis.

Debate over the phylogenetic relationships within Lemuriformes during the past decade has centered on the relationships of the four families Lemuridae, Lepilemuridae, Indriidae, and Cheirogaleidae to one another, with Daubentoniidae generally recognized as the basal lineage. The topology of our tree strongly supports the monophyly of the infraorder Lemuriformes, with 10 shared insertions recovered in support of this node. We recovered six loci placing Daubentoniidae as the most basal lineage among the five families. The position of Daubentoniidae is unsurprising in light of earlier studies, which established the aye-aye as basal to the other lemuriformes [3], [4], [17], [60]. The other four families segregate into a Lepilemuridae – Cheirogaleidae and an Indriidae – Lemuridae clade. Each of these clades is supported by a single insertion locus, MmM97 (GenBank accession numbers JX195194 and JX195195) and LI1 (GenBank accession numbers AY441631-AY441638), respectively. Cloning and sequencing of MmM97 confirmed that these two insertions are in fact the same element. LI1 was previously sequenced and confirmed by Roos et al. (2004) [3] with identical results. Other studies have supported a grouping of Lepilemuridae and Cheirogaleidae as sister taxa [17], [18]. The grouping of Lemuridae and Indriidae as sister taxa is also supported by previous studies [3], [15], [23], [24].

Support for the monophyly of the Lemuridae was recovered from 10 insertions. *Lemur* and *Hapalemur* were determined to be sister taxa, a position supported by six loci. A further eight loci support a *Lemur*-*Hapalemur* clade as sister to *Eulemur*, with *Varecia* sister taxa to the other three genera. These findings support the established phylogeny of lemurid taxonomy (*e.g.* [3], [7], [17], [18], [21]), in particular, the taxonomic separation of *Eulemur* from *Lemur* [61]. We also recovered three loci unique to *Lemur catta*, supporting the established convention that *Lemur catta* is the sole species in the genus *Lemur* [1], [18].

In *Eulemur* we recovered strong support for the unity of the *macaco* group (*Eulemur macaco* and *Eulemur flavifrons*, formerly identified as subspecies of *Eulemur macaco* and now elevated to full species status) to the exclusion of other members of the genus. We were unable to further elucidate relationships among the other species definitively, particularly within the closely related and monophyletic *fulvus* group. Groves (2001) [23] elevated the former subspecies in the *fulvus* group to full species status, a position supported by morphological and genetic evidence [62], [63], [64]. The genus *Eulemur*, created by Simons and Rumpler (1988) [60] to house the “true” lemurs when they were removed from *Lemur* after the species *Lemur catta* was designated the sole occupant of that genus, is believed to have diversified from a common ancestor over a relatively rapid span of time beginning ∼8mya. This rapid speciation was possibly driven by a wetter climate and changing plant life in Madagascar [17], [65], [66]. The recent divergence times within this genus likely contributed to the difficulty of identifying species-specific *Alu* elements.

The relationships between the 12 *Eulemur* species currently recognized [1] and whether all of them should be accorded full species status or remain subspecies, has been debated at length [8], [67]. Further confounding relationships and pointing to possibly overzealous species description in *Eulemur* are instances of observed hybridization between described species [63], [68], [69]. We chose to follow Mittermeier et al. (2010) [1] in recognizing each of the nine taxa available for our study (see Table 1) as full species, though we were unable to obtain lineage-specific *Alu* elements to support this hypothesis at the individual species level. We have opted to use the binomial identification for six of the species that were previously delineated as subspecies, for instance, *Eulemur albifrons* instead of *Eulemur fulvus albifrons*. A total of six shared insertion loci affirm the relationship between the two species of the *macaco* group and the four species of the *fulvus* group to the exclusion of the other three *Eulemur* species on our panel. An additional two loci were identified as being specific to *Eulemur macaco*, and may prove useful in the future as markers for species identification.

Within the family Cheirogalidae we recovered a strongly supported sister-group relationship between *Microcebus* and *Mirza,* with *Cheirogaleus* recovered as the basal lineage. A total of 19 loci supported the *Microcebus*-*Mirza* grouping to the exclusion of *Cheirogaleus*, which supports the findings of earlier phylogenetic studies [3], [17], [18], [70], [71]. While we were only able to obtain samples from *Microcebus murinus* for our study, 18 species are currently recognized in *Microcebus* [1]. The 16 *Microcebus*-specific loci identified in this study might be useful in future analyses to clarify relationships within this speciose genus. One or more of the *Alu* elements we identified could certainly be polymorphic between species in this genus, something we were unable to clarify with only a single species on our panel.

In the Indriidae clade we recovered eight loci. Two of these loci, PcC1 and PcC2, were taken from nuclear DNA sequence available via GenBank and are present in all four Indriidae species represented in our dataset. Among the other six loci, three (MmA2c, MmA20A, and Str67A) were obtained from the sequencing of ambiguous loci and the remaining three were taken from Roos et al. (2004) [3]. Of these six loci, four are present in all four Indriidae species examined in our study. One locus, MmA2c, (GenBank accession number JX195193) is specific to *Avahi laniger*, the eastern wooly lemur.

While *Alu*-based phylogenies are generally reliable, confounding events can occur that result in incongruent tree topologies [41], [42], [43]. In this case it is necessary to resolve relationships between species by DNA sequencing and comparative analysis of the element in question to establish the precise nature of a given locus. An example of a confounding event in the form of a parallel independent insertion is locus MmA20 (GenBank accession numbers JX195187-JX195189), which appeared to group the Cheirogaleidae with the Indriidae to the exclusion of the other species on our panel. This did not agree with the topology of our tree. Sequencing of this locus in both families demonstrated the presence of a near-parallel independent insertion event, with two *Alu* elements from independent subfamilies present at nearly the same location in the genome in the two different genera, that is, within the amplicon produced by the primers designed for this locus. MmA20 was then scored as Cheirogaleidae-specific, and MmA20A was scored as Indriidae-specific. Other loci found to contain parallel independent insertions include MmA2, M11, Str67A, and LcC2. Additionally, Ray et al. (2005) [42] present an excellent illustrating of potentially confounding *Alu* insertion events in their study of platyrrhine primate phylogeny.

### Conclusions

The robust phylogenetic relationships presented in this study support existing morphological and genetic research about relationships at the species and genus levels within the infraorder Lemuriformes. We offer support for a resolution of the previously unresolved relationships between the four families Lemuridae, Indriidae, Cheirogaleidae, and Lepilemuridae with a statistically robust tree (HI = 0.000) demonstrating that Daubentoniidae is the basal lineage among Lemuriformes, with the common ancestor of the remaining families later separating into Lemuridae – Indriidae and Cheirogalidae – Lepilemuridae clades. Additionally, we largely resolve the branching patterns within the Cheirogaleidae and Lemuridae families. The methods used to examine these relationships further affirm the strengths of SINE-based phylogenetic studies. Given the known proliferation of *Alu* elements found in primate genomes during previous studies we expect that these primate synapomorphies will continue to be useful phylogenetic tools in the future.

## Supporting Information

Table S1 **A table listing all primers used in this study with optimal annealing temperatures and source.** Primers highlighted in green were designed specifically for this study. Primers highlighted in yellow were designed by Herke et al. (2007). Primers highlighted in red were designed by Roos et al. (2004).
(XLSX)

Table S2 **A table showing the character matrix of amplification patterns of all loci in all species.** A “1” indicates the locus is a filled site (*Alu* element present) and a “0” indicates the locus is an empty site (*Alu* element absent) in the corresponding species. A “?” indicates no amplification occurred of that primer pair in that species.
(XLSX)
