# Correction: An *Alu*-Based Phylogeny of Lemurs (Infraorder: Lemuriformes)

**DOI:** 10.1371/annotation/378aaec2-599e-4cd4-8941-3a14c208b757

**Published:** 2012-08-31

**Authors:** Adam T. McLain, Thomas J. Meyer, Christopher Faulk, Scott W. Herke, J. Michael Oldenburg, Matthew G. Bourgeois, Camille F. Abshire, Christian Roos, Mark A. Batzer

Due to an error introduced during the production process, some references are incorrectly cited throughout the article. These errors begin in Materials and Methods in the Computational Methodology section. In the second paragraph, the reference in the sentence "Elements identified by RepeatMasker in *Microcebus murinus* genomic contigs as members of an *Alu*L subfamily and >280 bp in length were compared to four non-lemuriform primate genomes, human (hg19), chimpanzee (panTro2), orangutan (ponAbe2), and rhesus macaque (rheMac2), using the BLAST-Like Alignment Tool (BLAT) available at http://genome.ucsc.edu" should actually be reference 54. Subsequently, all references following this reference that are numbered above 54 should actually be one number higher (I.E. reference 54 then becomes 55, 55 becomes reference 56, etc.). Reference numbers below 53 are correct. The sentence beginning "The genus Eulemur, created by Simons and Rumpler (1988)" should be reference 62. 

